# Pt-decorated nanoporous gold for glucose electrooxidation in neutral and alkaline solutions

**DOI:** 10.1186/1556-276X-6-313

**Published:** 2011-04-07

**Authors:** Xiuling Yan, Xingbo Ge, Songzhi Cui

**Affiliations:** 1 School of Chemistry and Chemical Engineering, Shandong University, Jinan 250100, China; 2 School of Chemistry and Bioscience, Ili Normal University, Xinjiang 835000, China

## Abstract

Exploiting electrocatalysts with high activity for glucose oxidation is of central importance for practical applications such as glucose fuel cell. Pt-decorated nanoporous gold (NPG-Pt), created by depositing a thin layer of Pt on NPG surface, was proposed as an active electrode for glucose electrooxidation in neutral and alkaline solutions. The structure and surface properties of NPG-Pt were characterized by scanning electron microscopy (SEM), transmission electron microscopy (TEM), X-ray powder diffraction (XRD), and cyclic voltammetry (CV). The electrocatalytic activity toward glucose oxidation in neutral and alkaline solutions was evaluated, which was found to depend strongly on the surface structure of NPG-Pt. A direct glucose fuel cell (DGFC) was performed based on the novel membrane electrode materials. With a low precious metal load of less than 0.3 mg cm^-2 ^Au and 60 μg cm^-2 ^Pt in anode and commercial Pt/C in cathode, the performance of DGFC in alkaline is much better than that in neutral condition.

## Introduction

Glucose is widely used in modern life and industry as a nontoxic, inexpensive, and renewable resource. Since Rao and Drake [1] first reported the glucose oxidation on platinized-Pt electrodes in phosphate buffer solution in the 1960s, electrocatalytic oxidation of glucose has been extensively investigated as a key reaction in the fields of sensors [2,3] and fuel cells [4,5]. Great efforts have been made to develop catalytically active electrode materials for this reaction in the past two decades. As one of the most studied electrocatalyst, Pt was found to exhibit considerable activity for glucose oxidation at a negative potential in neutral and alkaline solutions [6]. However, systematical study showed that this electrocatalytic process was subject to serious poisoning due to adsorbed intermediates from the oxidation of glucose [7]. To mitigate the poisoning effect, Pt-based bimetallic catalysts such as Pt-Pb [8,9], Pt-Ru [10,11], and Pt-Au [4,12], have been developed to improve the electrocatalytic activity and selectivity. On the other hand, it is increasingly realized that glucose electrooxidation is sensitive to surface structure of the electrocatalyst. For example, Adzic et al. found that this reaction strongly depended on the crystallographic orientation of the Pt electrode surface [13]. Thus, significant attention has been focused on exploiting the potential applications of the nanostructured materials with special surface properties for glucose oxidation. Besides the widely used nanoparticles [14,15], many other nanostructures were also studied, such as carbon nanotubes [16], ordered Pt nanotube arrays [17], mesoporous Pt electrodes [18], and nanoporous Pt-Pb and Pt-Ir networks [8,19]. While these unique nanostructures exhibited considerable advantages as compared to traditional electrodes, they were mainly employed for glucose electrochemical detection. Exploiting nanostructures for potential applications in glucose fuel cell is still highly desirable.

Recently, Erlebacher and co-workers reported an interesting type of membrane electrode materials called nanoporous gold (NPG) leaves which could be made by chemically etching the white gold (AgAu alloy) leaves in corrosive medium [20]. Coupled with surface functionalization with other catalytically active material, such as Pt, the 100-nm-thick high surface area electrode materials demonstrated superior activities toward a series of important electrochemical reaction including methanol oxidation [21,22] and formic acid oxidation [23]. Preliminary studies also proved they could work as promising electrocatalysts in proton exchange membrane fuel cells at ultra-low Pt loading [24,25]. Here, we focus on their electrocatalytic properties toward glucose oxidation and its application in alkaline glucose fuel cells.

## Experimental

### Reagents and apparatus

All chemicals were of analytical grade and used as purchased without further purification. D-Glucose, NaOH, HNO_3 _(65%), Na_2_HPO_4_·12H_2_O, NaH_2_PO_4_·2H_2_O, and H_2_PtCl_6_·6H_2_O were obtained from Sinopharm Chemical Reagent Co., Ltd. Au/Ag alloy (50:50, wt%) leaves with thickness of 100 nm (Sepp Leaf Products, New York) were used for NPG fabrication. Ultrapure water (18.2 MΩ) was used throughout the experiments and 0.1 M PBS was prepared with pH 7.4. The composition of NPG-Pt sample was determined by an IRIS Advantage inductively coupled plasma-atomic emission spectrometry (ICP-AES). The surface structure of NPG-Pt was observed JSM-6700F SEM and JEM-2100 TEM. The crystallographic information was obtained with XRD (Bruker D8 Advance X-ray diffractometer, Cu Kα radiation λ = 1.5418 Å) at a 0.02°/s scan rate. All electrochemical measurements were performed at room temperate in a traditional three-electrode electrochemical cell with a CHI 760C electrochemical workstation (Shanghai). Mercury sulfate electrode (MSE) was selected as reference electrode in all the electrochemical measurements, and a pure Pt foil as the counter electrode. Both PBS and the mixed solutions were purged with high pure nitrogen (99.999%) for 30 min prior to measuring.

Membrane electrode assembly (MEA) was prepared by attaching NPG-Pt to carbon paper (TGP-H-060, Toray, Japan) first, and then hot-pressed onto one side of a Nafion 115 membrane and commercial Pt/C (60 wt%, Johnson Matthey, UK) onto another side at 110°C and 1.5 MPa for 195 s. As-prepared MEAs were then assembled between high purity graphite plates as flow and current collecting plates, which have single channel serpentine flow pattern. The anolyte was pumped to anode by peristaltic pump, while pure oxygen was fed to the cathode without humidification by a massflow controller. The cell temperature was controlled through a temperature controller and monitored by thermocouples buried in the graphite blocks. The steady state polarization curves were recorded by automatic Electric Load (PLZ 70UA, Japan).

### Preparation of NPG and NPG-Pt electrodes

NPG was made by dealloying commercial 12-carat white gold membrane in concentrated nitric acid for 20 min at 30°C [20]. Subsequently, NPG were immediately transferred to ultrapure water and repeatedly washed to remove Ag^+ ^and NO_3_^-^. NPG-Pt samples were prepared by floating the as-prepared NPG membranes at the interface between the H_2_PtCl_6 _(1 g/L, pH = 10) solution and the vapor of hydrazine hydrate (85%) in a closed system [22]. Deposition reaction occurred uniformly on the surface of NPG. The amount of Pt deposited onto the NPG substrate gradually accumulates with increasing plating time. The as-prepared NPG-Pt (loading of 0.1 mg cm^-2 ^Au and 20 μg cm^-2 ^Pt) samples were transferred into ultrapure water as soon as the plating reaction finished. Then NPG-Pt membranes were affixed onto the clean GC electrode (4 mm in diameter) and fixed with 2 μL dilute nafion solution (0.5 wt%). The as-prepared NPG-Pt electrode was dried at room temperature for 24 h before measurements.

## Results and discussion

### Surface and crystal structure of the NPG-Pt

**Figure 1:**
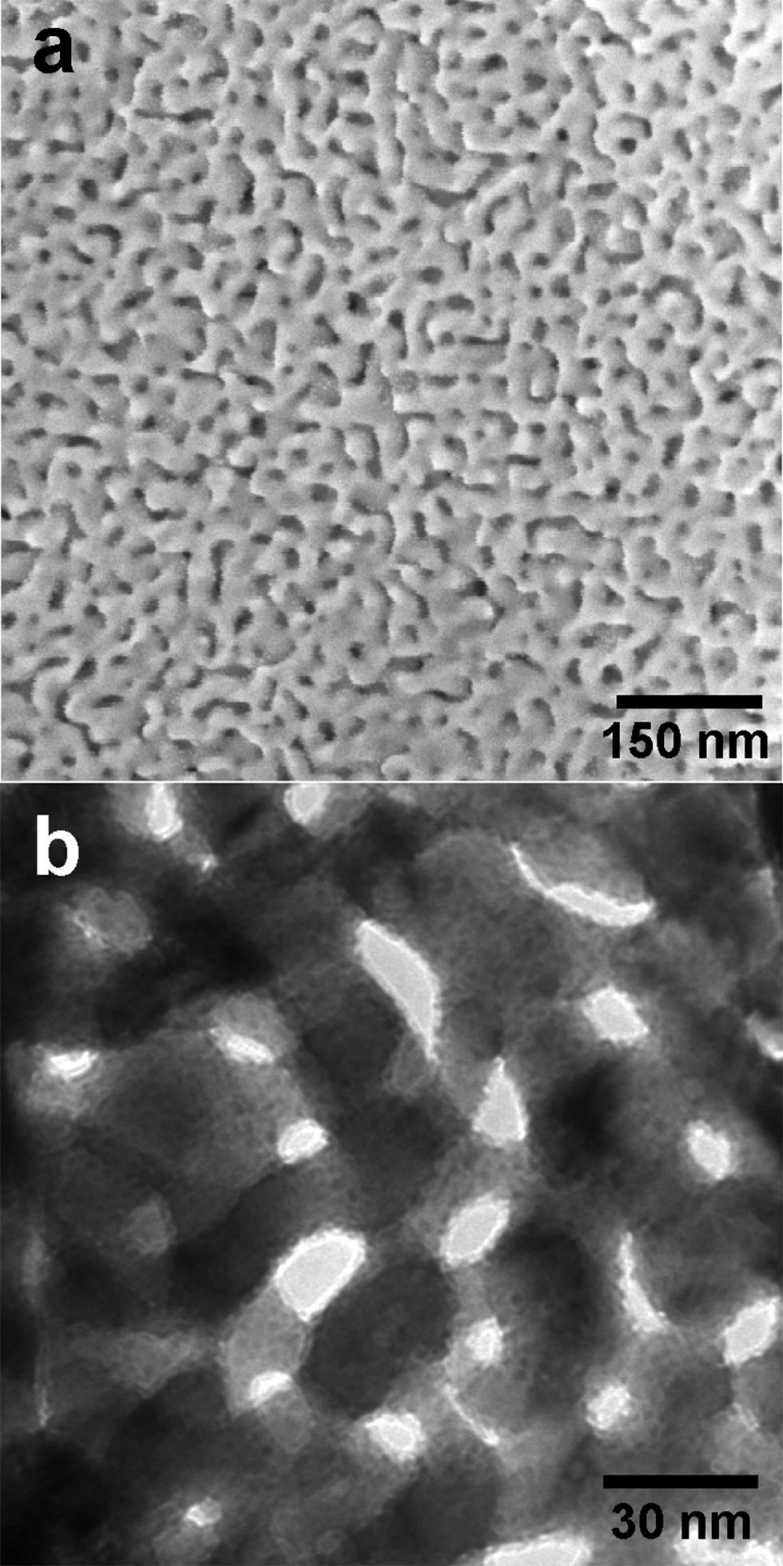
**Typical SEM **(a) **and TEM **(b) **images of the NPG-Pt 64 sample**.

NPG-Pt samples were fabricated by chemical plating a thin layer of Pt on NPG ligament surfaces. Figure 1a shows the wide scan SEM image of the as prepared NPG-Pt, which exhibits a three-dimensional continuous nanoporous structure, similar to the reported NPG [20]. Such structure is highly desirable in electrocatalysis because of its structural integrity and electron conductivity. TEM observation (Figure 1b) clearly reveals that for heavily plated samples, the deposited Pt form nanoislands uniformly coating on NPG surface. Previous studies have proved that these Pt islands adopt a conformal and epitaxial relationship to the NPG substrate [24]. The amount and size of the Pt islands are controlled by varying the reaction time. According to ICP-AES results, plating for 8 and 64 min (signed as NPG-Pt 8 and NPG-Pt 64, respectively) resulted in a Pt loading of approximately 6 and 20 μg cm^-2 ^in the final products, respectively.

**Figure 2:**
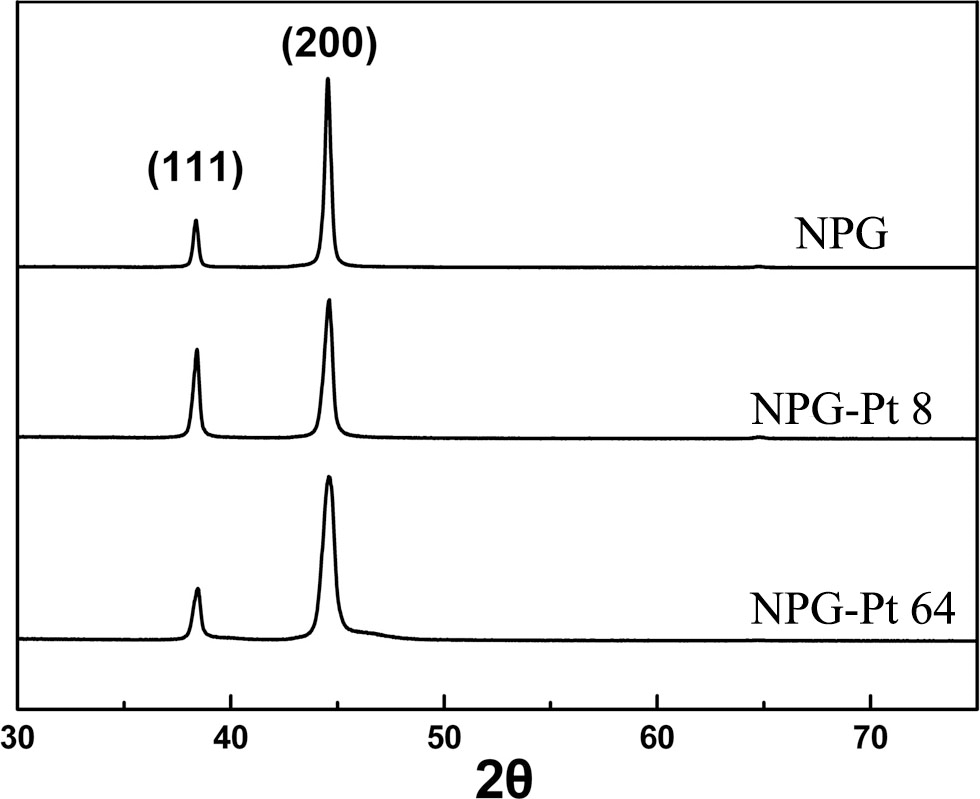
**XRD patterns for NPG, NPG-Pt 8 and NPG-Pt 64 samples**.

XRD was employed to investigate the crystalline structure of NPG-Pt. Figure 2 shows XRD patterns from NPG and NPG-Pt samples which nearly exhibit the same patterns. The diffraction peaks at 2*θ *= 38.4°, 44.5° can be ascribed to the (111), (200) planes of face-centered cubic Au crystals respectively, with a lightly positive shift relative to standard pattern. This common positive shift of diffraction peaks are believed to result from the strain in the nanoporous structure [26]. Interestingly, the (200) peak exhibits a much higher intensity than the theoretical value and even exceeds the (111) peak, while (220) peak is nearly invisible in the patterns. These behaviors suggest that Pt plating does not affect the texture of the NPG membranes. Pt surface layer would not be able to exhibit its distinct diffractions due to its extremely low existing amount.

### Electrochemical characteristics of NPG-Pt in PBS

**Figure 3:**
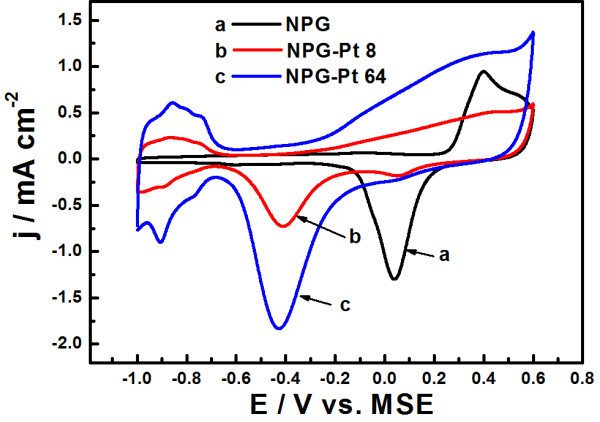
**CV curves for NPG and NPG-Pt 8, NPG-Pt 64 samples in 0.1 M PBS, scan rate: 50 mV s^-1^**. The currents were normalized to the geometrical areas.

The NPG-Pt electrodes were further characterized by means of CV in 0.1 M PBS, as shown in Figure 3, where NPG was also included for comparison. The fresh NPG exhibits an obvious anodic current rise at approximately 0.4 V and a sharp cathodic peak at approximately 0.05 V for Au surface oxides formation and reduction, respectively, similar to the reported polycrystalline Au electrode in PBS [27]. After plating, it could be observed that the well-defined hydrogen adsorption/desorption peaks in the potential region between ~ -1.0 and -0.7 V show up and gradually increase in intensity with the plating time. The Pt surface oxides formation begins at approximately 0.2 V and the corresponding oxides reduction peaks appear at approximately -0.42 V. Meanwhile, the signals for gold surface oxides formation and reduction nearly disappear in the entire potential range, indicating a near complete coverage by the deposited Pt. These electrochemical characteristics of NPG-Pt are in good agreement with previous observations in acid solutions [22].

### Electrocatalytic properties of NPG-Pt for glucose oxidation in neutral and alkaline solutions

**Figure 4:**
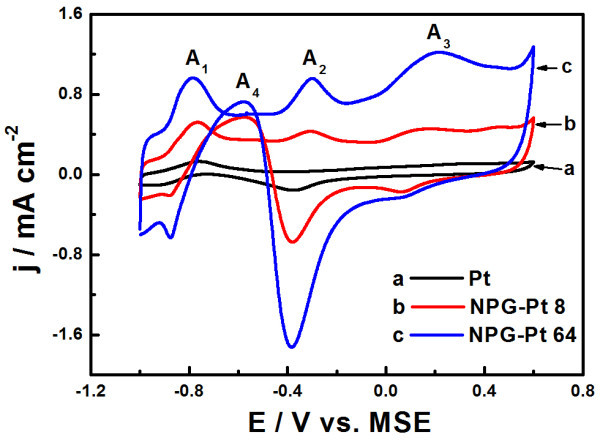
**CV curves obtained for NPG-Pt 8 and NPG-Pt 64 samples in a mixed solution of 0.1 M PBS + 10 mM glucose, scan rate: 50 mV s^-1^**. Pure Pt electrode was included for comparison and the currents were normalized to the geometrical areas.

The electrocatalytic activity of NPG-Pt toward glucose oxidation was evaluated by CV in PBS containing 10 mM glucose, and a pure Pt electrode with smooth surface was also included for comparison. As shown in Figure 4, all three samples show similar voltammetric behavior in the presence of glucose, i.e., three main oxidation peaks (A_1_, A_2_, and A_3_) appear during the positive potential scan at -0.84, -0.3, and 0.2 V, respectively, similar to the glucose oxidation on Pt-rich Au-Pt alloy nanoparticles [4]. The peak A_1 _at the low potential region is often attributed to the dehydrogenation of glucose on active Pt surface, producing a layer of adsorbed glucose intermediates on electrode surface [8]. These intermediate species were then oxidized at a positive potential, resulting in peaks A_2 _and A_3_. Further increasing the potential, surface metal oxides generate which are nearly inactive for glucose oxidation, resulting in a current drop at higher potential. The peak A_4 _was ascribed to the glucose electroadsorption on the freshly produced active Pt surface at approximately -0.4 V during the negative scan. These voltammetric feathers are also similar to other reported Pt-based bimetallic electrode, reflecting a similar reaction process. Meanwhile, it is observed that NPG-Pt samples exhibits substantially higher peak current densities than Pt electrode, indicating a superior catalytic activity toward glucose oxidation. In addition, NPG-Pt 64 exhibits the highest activity among the three samples, due to the largest active surface area as revealed by CV in PBS in Figure 3. It is noted that NPG-Pt membrane can directly be used as an unsupported electrocatalyst in PEM fuel cells [24,25]; therefore, these unique nanostructures can be expected to function as active bimetallic anode catalysts in glucose fuel cells.

**Figure 5:**
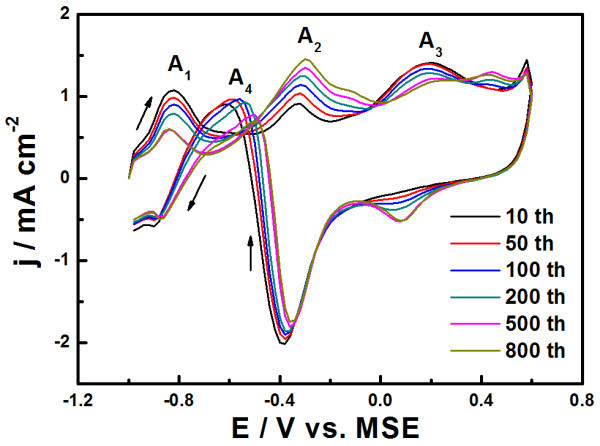
**Prolonged CV curves of NPG-Pt 64 electrode in PBS containing10 mM glucose, scan rate: 50 mV s^-1^**. The currents were normalized to the geometrical areas.

In order to gain further insight into the surface structure effect of NPG-Pt on catalytic performance in glucose oxidation, the prolonged CV tests up to 800 cycles were conducted on NPG-Pt 64 sample. In this electrochemical process, the surface composite and structure would be substantially changed by the repeated redox of the surface metal. This structure change was also found to strongly affect the catalytic properties of NPG-Pt, as shown in Figure 5. While the peaks A_1 _and A_3 _gradually decrease with the CV cycles, peak A_2 _obviously increases in intensity and the onset potential also lightly shifts to a negative value. According to the above discussion, the loss of active Pt surface, resulting from the surface Pt alloying with the NPG substrate during the CV process, would be responsible for the corresponding peak decrease for A_1 _and A_3_. Meanwhile, the peak A_2 _expansion suggests that the new surface from CV process is more active for the intermediate species. This is not surprised since Au is active for glucose oxidation at this potential in PBS [27]. Therefore, we could improve the catalytic performance of NPG-Pt by tailoring the surface structure to maintain the catalytic activity at low potential and enhance the ability of oxidizing the adsorbed intermediate species (because these intermediate can hinder the glucose adsorption on Pt surface).

**Figure 6:**
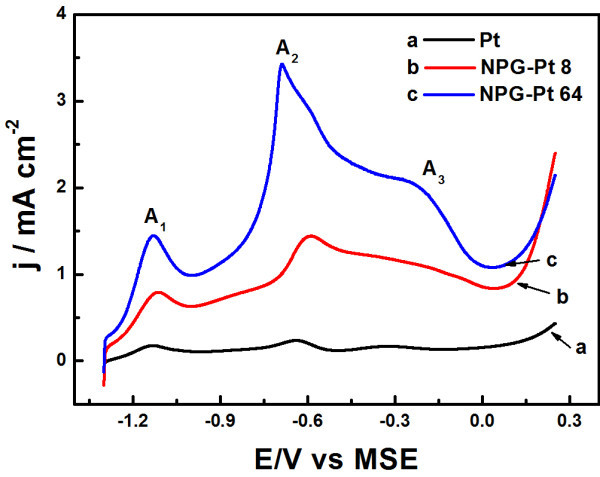
**CV curves for NPG-Pt 8 and NPG-Pt 64 samples in a mixed solution of 0.1 M NaOH + 10 mM glucose, scan rate: 50 mV s^-1^**. Pure Pt electrode was included for comparison and the currents were normalized to the geometrical areas.

Figure 6 shows the CV curves of NPG-Pt in the mixed solution of NaOH and 10 mM glucose. As in PBS, three oxidation peaks were observed in the positive scan, indicating a similar reaction process. Nevertheless, the observed high current densities as compared to that in PBS suggest that glucose oxidation in alkaline solution proceeds more rapidly than in neutral solution, due to the high concentration of OH^- ^ions which are believed to be directly involved in the reaction intermediates oxidation [6]. This is also in agreement with previous observation that Pt-decorated NPG could exhibit high activity and good stability for methanol oxidation in alkaline solution [21]. Again, the NPG-Pt 64 sample exhibits the highest activity, with a peak current density approximately 1.5 and 3.4 mA cm^-2 ^for peaks A_1 _and A_2_, respectively, which are about seven times higher than those on pure Pt electrode.

### DGFCs in neutral and alkaline solution

**Figure 7:**
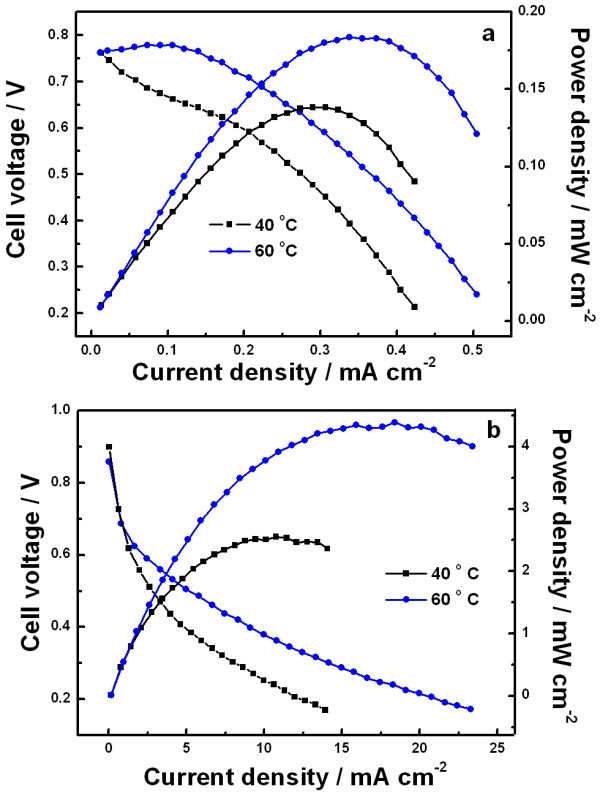
**Performance of DGFC at various temperatures in 0.1 M PBS containing 0.5 M glucose (a) and in 2 M NaOH containing 0.5 M glucose (b) with NPG-Pt 64 as the catalyst for anode and commercial Pt/C as cathode**. The flow rates of the anolyte and the air are 2 and 120 mL min^-1^, respectively.

Figure 7 shows typical polarization curves of DGFC with NPG-Pt 64 working as anode and commercial Pt/C as cathode catalyst, and Nafion 115 membrane as electrolyte at 40 and 60°C in neutral and alkaline solutions. The loading of the catalyst were 0.3 mg cm^-2 ^Au and 60 μg cm^-2 ^Pt which are three times as much as those in previous experiment. The OCVs (Figure 7a) were almost the same (~0.8 V) at 40 and 60°C and their maximum power densities were 0.14 and 0.18 mW cm^-2^, which was much higher than the one reported [28]. In alkaline condition (Figure 7b), the OCVs were almost the same too (~0.9 V) at 40 and 60°C and accordingly their maximum power densities were 2.5 and 4.4 mW cm^-2^, which exceed the reported data [29,30]. By maintaining the concentration of glucose at 0.5 M in 0.1 M PBS and 2 M NaOH respectively, it can be observed that both in neutral and alkaline solutions, the cell performance increased with temperature, which would be due to the faster electrochemical kinetics of both the anodic and cathodic reactions, increased conductivity of the electrolyte and enhanced diffusion rate of glucose and oxygen.

It also can be seen that the maximum power densities in alkaline (Figure 7b) was 4.4 mW cm^-2 ^which is about 24 times than that in neutral solution (0.18 mW cm^-2^, Figure 7a). This should be mainly attributed to quicker reaction rate on the NPG-Pt in alkaline than that in neutral solution for glucose oxidation which was in line with the results of 3.3 above.

## Conclusions

NPG-Pt membranes, a type of porous Au-Pt bimetallic nanostructures, were fabricated by chemically plating thin layer of Pt on NPG and were studied for glucose electrooxidation and the application in fuel cell. Taking advantage of the unique structure and high surface area, NPG-Pt exhibits considerable activity toward this reaction in neutral and alkaline solutions. In addition, glucose oxidation on NPG-Pt was found to be a surface sensitive process and Au-Pt surface alloy is highly active for oxidizing the adsorbed intermediate species resulted from the glucose electroadsorption. This means we could further improve the catalytic performance of NPG-Pt by tailoring the surface composite and structure. The results of DGFC test indicated that NPG-Pt is expected as a promising low precious metal loading electrocatalyst for application in glucose fuel cells.

## Abbreviations

CV: cyclic voltammetry; DGFC: direct glucose fuel cell; ICP-AES: inductively coupled plasma-atomic emission spectrometry; MEA: membrane electrode assembly; MSE: mercury sulfate electrode; NPG: nanoporous gold; NPG-Pt: Pt-decorated nanoporous gold; SEM: scanning electron microscopy; TEM: transmission electron microscopy; XRD: X-ray powder diffraction.

## Competing interests

The authors declare that they have no competing interests.

## Authors' contributions

Songzhi Cui carried out the electrochemical measurements and drafted the manuscript. Xinbo Ge carried out the XRD studies, participated in the sequence alignment and revised the manuscript. Xiuling yan conceived of the study, and participated in its design and performed the fuel cell tests. All authors read and approved the final manuscript.
